# Right-sided versus left-sided colorectal cancer in elderly patients: a sub-analysis of a large multicenter case–control study in Japan

**DOI:** 10.1007/s00595-024-02827-9

**Published:** 2024-06-05

**Authors:** Haruki Sada, Takao Hinoi, Hiroaki Niitsu, Hideki Ohdan, Seiichiro Yamamoto, Shungo Endo, Koya Hida, Yusuke Kinugasa, Toshiyuki Enomoto, Satoshi Maruyama, Fumio Konishi, Masahiko Watanabe, The Japan Society of Laparoscopic Colorectal Surgery, Eiji Kanehira, Kunihisa Shiozawa, Hiroyuki Bando, Daisuke Yamamoto, Seigo Kitano, Masafumi Inomata, Tomonori Akagi, Junji Okuda, Keitaro Tanaka, Masayoshi Yasui, Kosei Hirakawa, Kiyoshi Maeda, Akiyoshi Kanazawa, Junichi Hasegawa, Junichi Nishimura, Shintaro Akamoto, Masashi Ueno, Hiroya Kuroyanagi, Masaki Naito, Takashi Ueki, Yoshiharu Sakai, Koya Hida, Yousuke Kinjo, Yukihito Kokuba, Madoka Hamada, Norio Saito, Masaaki Ito, Shigeki Yamaguchi, Jou Tashiro, Toshimasa Yatsuoka, Tomohisa Furuhata, Kenji Okita, Yoshiro Kubo, Shuji Saito, Yosuke Kinugasa, Fumio Konishi, Kazuhiro Sakamoto, Michitoshi Goto, Junichi Tanaka, Nobuyoshi Miyajima, Tadashi Suda, Tsukasa Shimamura, Yoshihisa Saida, Toshiyuki Enomoto, Takeshi Naito, Yasuhiro Munakata, Ken Hayashi, Yasukimi Takii, Satoshi Maruyama, Yohei Kurose, Yasuhiro Miyake, Shoichi Hazama, Shoich Fujii, Shigeru Yamagishi, Masazumi Okajima, Seiichiro Yamamoto, Hisanaga Horie, Kohei Murata, Kenichi Sugihara

**Affiliations:** 1 Department of Surgery, Kure Medical Center and Chugoku Cancer Center, 3-1 Aoyama-Cho, Kure, Hiroshima 737-0023 Japan; 2 Department of Clinical and Molecular Genetics, Hiroshima University Hospital, 1-2-3 Kasumi, Minami-Ku, Hiroshima, 734-8551 Japan; 3 Division of Molecular Therapeutics, Aichi Cancer Center Research Institute, 1-1 Kanokoden, Chikusa-Ku, Nagoya, Aichi 464-8681 Japan; 4 Department of Gastroenterological and Transplant Surgery, Graduate School of Biomedical and Health Sciences, Hiroshima University, 1-2-3 Kasumi, Minami-Ku, Hiroshima, 734-8551 Japan; 5 Department of Gastroenterological Surgery, Tokai University School of Medicine, 43 Shimokasuya, Isehara, Kanagawa 259-1103 Japan; 6 Department of Coloproctology, Aizu Medical Center, Fukushima Medical University, Aizu-Wakamatsu City, Fukushima 969-3492 Japan; 7 Department of Surgery, Kyoto University Hospital, Kyoto, Japan; 8 Department of Gastrointestinal Surgery, Tokyo Medical and Dental University, Tokyo, Japan; 9 Department of Surgery, Toho University Ohashi Medical Center, 2-22-36 Ohashi, Meguro-Ku, Tokyo, Japan; 10 Department of Gastrointestinal Surgery, Niigata Cancer Center Hospital, 2-15-3 Kawagishicho, Niigata, 951-8566 Japan; 11 Department of Surgery, Nerima Hikarigaoka Hospital, 2-11-1 Hikarigaoka, Nerima-Ku, Tokyo, 179-0072 Japan; 12 Department of Surgery, Kitasato University Kitasato Institute Hospital, Shirokane 5-9-1, Minato-Ku, Tokyo, 108-8642 Japan

**Keywords:** Colorectal cancer, Elderly patients, Sidedness, Laparoscopic, Open

## Abstract

**Purpose:**

This study investigated the impact of sidedness of colorectal cancer (CRC) in elderly patients on the prognosis.

**Methods:**

In a sub-analysis of a multicenter case–control study of CRC patients who underwent surgery at ≥ 80 years old conducted in Japan between 2003 and 2007, both short- and long-term outcomes were compared between right-sided colon cancers (RCCs) and left-sided colorectal cancers (LCCs). RCCs were defined as those located from the cecum to the transverse colon.

**Results:**

Among the 1680 patients who underwent curative surgery, 812 and 868 had RCCs and LCCs, respectively. RCCs were more frequent than LCCs in those who were female, had renal comorbidities, and had a history of abdominal surgery. Regarding tumor characteristics, RCCs were larger, invaded more deeply, and were diagnosed as either mucinous or signet ring-cell carcinoma more frequently than LCCs. Regarding the prognosis, patients with RCCs had a significantly longer cancer-specific survival (CS-S) and cancer-specific relapse-free survival (CS-RFS) than those with LCCs. Furthermore, sidedness was determined to be an independent prognostic factor for CS-S and CS-RFS.

**Conclusion:**

RCCs, which accounted for half of the cases in patients ≥ 80 years old, showed better long-term outcomes than LCCs.

**Supplementary Information:**

The online version contains supplementary material available at 10.1007/s00595-024-02827-9.

## Introduction

Colorectal cancer (CRC) is a leading cause of cancer-related deaths worldwide [1, 2], and its incidence is expected to increase with the rapid aging of society. In general, the risk of postoperative complications is higher among elderly patients who undergo surgery than among younger patients because elderly patients tend to have more preoperative comorbidities and are frailer than younger patients. Therefore, safe and effective treatment of elderly patients with CRC is a challenge that must be met [3]. However, whether or not laparoscopic surgery is beneficial and safe in such patients remains unclear.

Although laparoscopic surgery has the advantage of facilitating early recovery after surgery in the general population, its long operation time, use of pneumoperitoneum, and extreme head-down position during laparoscopic surgery remain concerns, as these affect circulatory dynamics and perioperative morbidity rates among the elderly. To address this issue, we conducted a multicenter observational study of 2065 elderly patients ≥ 80 years old who had undergone CRC surgery and demonstrated the safety of laparoscopic surgery with comparable long-term outcomes to open surgery, with the identification of some risk factors affecting short- and long-term outcomes [3–10].

It was recently revealed that metastatic CRCs originating from the right-sided colon have a lower survival rate than those originating from the left-sided colon [11]. Similarly, other studies have shown that right-sided colon cancers (RCCs) have different prognoses from left-sided colorectal cancers (LCCs) [12–25]. Therefore, the location of the primary tumor is now recognized as a prognostic factor.

Most recently, many translational studies have elucidated that the different prognoses of RCCs and LCCs partially depend on the biological characteristics of the tumor, defined by molecular aberrations, including microsatellite instability (MSI) [12, 14, 15, 23, 26–31]. MSI-high CRCs are associated with a lower rate of distant or lymph node metastases [17, 26, 32] and lower recurrence rates after curative surgery, resulting in a longer survival after curative surgery than microsatellite stable CRCs [12, 26–28, 33]. However, once distant metastases develop, they are refractory due to resistance to 5-FU-based chemotherapy [28, 34–37].

MSI-high CRCs are also more frequently observed in cases of RCC [19, 26, 27] and in the elderly than in other patients [27]. From this standpoint, RCCs are expected to have a better prognosis after curative surgery than LCCs in the elderly population. Currently, evidence regarding whether RCCs or LCCs exhibit a better prognosis after surgery in elderly patients is lacking. A surveillance program and treatment strategy among elderly patients after surgery for CRC that considers surgical complications and prognoses based on differences in tumor location should be established. However, little is known regarding the impact of tumor location on short- and long-term outcomes after curative surgery for CRCs among the elderly.

In the present study, we analyzed a database from a large multicenter study focused on individuals ≥ 80 years old to elucidate the impact of CRC tumor location on survival outcomes among an elderly population.

## Methods

### Study design and participants

As described above, we previously reported a multicenter observational study of 2065 elderly patients ≥ 80 years old with a history of CRC surgery to assess the safety and efficacy of laparoscopic surgery [3–10]. Data were retrospectively collected between 2003 and 2007 from 41 institutes in the Japan Society of Laparoscopic Colorectal Surgery. In the present study, using this dataset in accordance with the approval of the Ethics Review Board of the Japanese Society for Cancer of the Colon and Rectum, we examined how the sidedness of CRCs influences the short-term surgical outcomes and survival among elderly patients ≥ 80 years old.

**Fig. 1:**
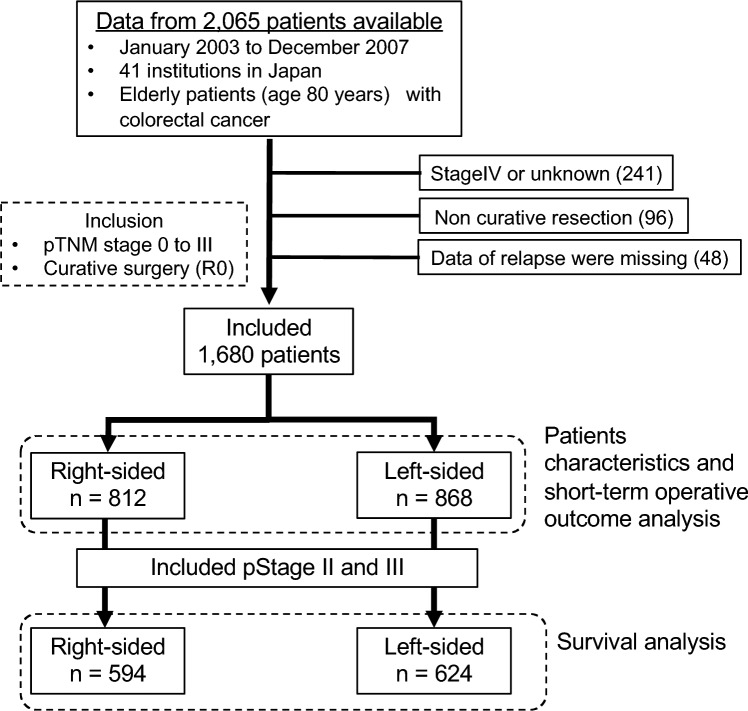
Patients and inclusion criteria for the data analysis relating to tumor location divided into right- and left-side cases for patients ≥ 80 years old in this study

From the original cohort, we included all patients who were histologically diagnosed with colorectal adenocarcinoma and treated with either open or laparoscopic surgery. Patients with metastatic CRC at the time of the diagnosis and those who underwent non-curative surgery were excluded. As surveillance after surgery was performed in accordance with the standard protocols of individual institutions owing to the retrospective nature of this observational study, cases with missing data on relapse were excluded. A total of 1,680 patients who underwent curative surgery for pathological TNM stage 0 to III CRC were included and divided into RCC and LCC groups based on their embryologic origin. Specifically, RCCs were defined by a location from the cecum to the splenic flexure (midgut), whereas LCCs were defined by a location from the descending colon to the rectum (hindgut). Patient characteristics and short-term operative outcomes were studied in patients with stage 0–III CRC, and a survival analysis was performed in patients with stage II and III CRC (Fig. 1).

### Statistical analyses

The following parameters were compared between the RCC and LCC groups: age, sex, body mass index (BMI), Eastern Cooperative Oncology Group Performance States Scale (ECOG-PS), American Society of Anesthesiology (ASA) class, preoperative comorbidity, history of abdominal surgery, Union for International Cancer Control (UICC) TNM staging system (UICC TNM 7th edition), tumor diameter, and histological type of the tumor. The following short-term outcomes were also compared: approach (open versus laparoscopic), surgical duration, blood loss, blood transfusion, postoperative complications, and length of hospital stay.

Regarding long-term outcomes, the overall survival (OS) and relapse-free survival (RFS) were compared between the RCC and LCC groups. In addition to the OS and RFS, we analyzed the cancer-specific survival (CS-S) and cancer-specific RFS (CS-RFS), as older CRC patients may die of old age or other severe comorbidities independently of post-operative complications or CRC. In the CS-S and CS-RFS analyses, patients with a cause of death other than CRC were right-censored. Subsequently, multivariate analyses for the OS, RFS, CS-S, and CS-RFS were conducted using a Cox proportional hazards model balanced with the following variables: tumor location, age, sex, BMI, ASA class, comorbidity, previous abdominal surgery, TNM stage, histological findings, and surgical approach. The results are reported as the median and interquartile range for quantitative variables and as frequencies for categorical variables.

Comparisons were conducted using Wilcoxon’s rank-sum test for quantitative variables and Fisher’s exact test (binary) or Pearson’s Chi-squared test (more than three variables) for categorical variables. Survival time analyses were summarized using Kaplan–Meier curves and corresponding hazard ratios (HRs) with 95% confidence intervals (CIs). The results of the multivariate analysis for the OS, RFS, CS-S, and CS-RFS are presented as odds ratios (ORs) or HRs and 95% CIs, with corresponding p-values.

All statistical analyses were performed using the Statistical Package Social Statistics software program for Windows (version 22.0; IBM Corp., Armonk, NY, USA).

### Analyzing The Cancer Genome Atlas (TCGA), Pancancer atlas, colorectal adenocarcinoma (COADREAD) dataset

The following parameters were extracted from the TCGA-Pancancer Atlas COADREAD dataset: age, sex, International Classification of Diseases for Oncology, 3rd Edition (ICD-O-3) site code, MSIsensor score, and MSI MANTIS score. The tumor location was identified by the ICD-O-3 site code, and for cases unidentifiable by ICD-O-3, manual curation was performed by referring to the anatomical neoplasm subdivision in the dataset. MSI-high was defined as an MSIsensor score ≥ 3.5 or MSI MANTIS score ≥ 0.4 [38].

## Results

### Baseline characteristics of patients and tumors

Among the 1680 patients included in this study, 812 (48.3%) and 868 (51.7%) had RCCs and LCCs, respectively. The tumor locations are described in detail in Supplementary Table 1. There were significantly more females in the RCC group than in the LCC group. In this cohort, the rates of renal comorbidity and a history of abdominal surgery were also higher in the RCC group than in the LCC group. In contrast, there were no significant differences in the age, BMI, PS, ASA class, or comorbidities between the RCC and LCC groups.

Regarding the TNM staging system, tumors were larger and more invasive (higher T stage) in the RCC group than in the LCC group, although there were no significant differences in TNM stage or lymph node metastasis. Of note, the ratios of mucinous adenocarcinoma and signet ring cell carcinoma in the historical findings were greater among RCCs than among LCCs (Table 1).

**Table 1:** The comparison of baseline characteristics in patients ≥ 80 years old between right- and left-sided colorectal cancer

	Right-sided (n = 812)	Left-sided (n = 868)	p-value
Sex, n (%)			
Male	353 (43.5%)	451 (52.0%)	< 0.01
Female	459 (56.5%)	417 (48.0%)	
Age (year), median (IQR)			
All	83 (81–85)	83 (81–85)	0.68
Male	83 (81–85)	83 (81–85)	0.71
Female	83 (81–85)	83 (81–85.5)	0.97
Body mass index, kg/mm^2^			
< 18.5	157 (19.7%)	169 (20.0%)	0.96
18.5 to < 25.0	513 (64.4%)	539 (63.7%)	
≥ 25.0	127 (15.9%)	138 (16.3%)	
Performance status, n (%)			
0	340 (43.2%)	345 (40.5%)	0.24
1	268 (34.0%)	286 (33.6%)	
2	134 (17.0%)	171 (20.1%)	
3	44 (5.6%)	42 (4.9%)	
4	2 (0.3%)	7 (0.8%)	
ASA-class, n (%)			
1	107 (13.5%)	111 (13.2%)	0.63
2	494 (62.4%)	544 (64.5%)	
3	187 (23.6%)	182 (21.6%)	
4	4 (0.5%)	7 (0.83%)	
Comorbidity, n (%)			
Overall	648 (79.8%)	671 (77.3%)	0.21
Cardiovascular	166 (20.4%)	147 (16.9%)	0.069
Pulmonary	368 (45.3%)	395 (45.5%)	0.94
Cerebrovascular	81 (10.0%)	81 (9.3%)	0.68
Hepatic	23 (2.8%)	25 (2.9%)	1.00
Renal	24 (3.0%)	13 (1.5%)	0.047
Diabetes mellitus	86 (10.6%)	119 (13.7%)	0.053
Dementia	13 (1.6%)	10 (1.2%)	0.53
Previous abdominal surgery, n (%)	304 (37.4%)	272 (31.3%)	< 0.01
TNM stage			
0	37 (4.6%)	31 (3.6%)	0.42
I	181 (22.3%)	213 (24.5%)	
II	356 (43.8%)	358 (41.2%)	
III	238 (29.3%)	266 (30.7%)	
Diameter, mm, median (IQR)	40 (28–64)	40 (28–55)	0.025
T factor, n (%)			
T0-1	131 (16.1%)	139 (16.0%)	0.04
T2	111 (13.7%)	143 (16.5%)	
T3	405 (50.0%)	378 (43.6%)	
T4	165 (20.3%)	208 (24.0%)	
N factor, n (%)			
N0	574 (70.1%)	602 (69.4%)	0.31
N1	183 (22.5%)	218 (25.1%)	
N2	55 (6.8%)	48 (5.5%)	
Number of metastatic nodes, median (IQR)	0 (0–1)	0 (0–1)	0.67
Histology			
Muc + Sig	36 (4.5%)	17 (2.0%)	< 0.01
Others	767 (95.5%)	844 (98.0%)	

Values are reported as the number and percentage or the median and interquartile range

*ASA* American Society of Anesthesiologists, *Muc + Sig* Mucinous adenocarcinoma and signet ring cell carcinoma, *IQR* interquartile range

### The comparison of short-term outcomes between RCCs and LCCs

The surgical approach (open versus laparoscopic) and blood transfusion performance were not significantly different between the RCCs and LCCs. Surgeries for LCCs took longer to perform, had more blood loss, and required longer hospital stays than surgeries for RCCs. There was a trend toward fewer postoperative complications in LCCs than in RCCs, although the difference was not statistically significant (Table 2). According to the multivariate analyses, female sex, early tumor stage, and laparoscopic approach were independently associated with a lower morbidity rate (Table 3).

**Table 2:** The comparison of short-term postoperative outcomes in patients ≥ 80 years old between right- and left-sided colorectal cancer

	Right-sided (n = 812)	Left-sided (n = 868)	p-value
Approach			
Open	521 (64.2%)	553 (63.7%)	0.88
Laparoscopic	291 (35.8%)	315 (36.3%)	
Surgical duration, min, Median (IQR)	160 (125–209)	191 (145–250)	< 0.01
Blood loss, g, Median (IQR)	55 (20–150)	100 (30–263)	< 0.01
Blood Transfusion, n (%)	77 (9.7%)	99 (11.7%)	0.20
Postoperative complication, n (%)			
Overall	262 (32.3%)	320 (36.9%)	0.051
Postoperative ileus	57 (7.0%)	57 (6.6%)	0.71
Superficial SSI	81 (10.0%)	85 (9.8%)	0.93
Deep/Organ SSI	9 (1.1%)	19 (2.2%)	0.089
Delirium	63 (7.8%)	84 (9.7%)	0.17
Pneumonia	19 (2.3%)	20 (2.3%)	1.00
Anastomotic leakage	10 (1.2%)	20 (2.3%)	0.14
Postoperative Bleeding	7 (0.9%)	12 (1.4%)	0.36
Cardiac event	8 (1.0%)	3 (0.4%)	0.13
Hospital Stay, days, Median (IQR)	13 (10–18)	15 (11–22)	< 0.01

Values are reported as the number and percentage or the median and IQR

*SSI* surgical site infection, *IQR* interquartile range

**Table 3:** Results of a multivariate analysis for morbidities as postoperative complications in patients ≥ 80 years old with colorectal cancer

	Odds ratio	95% CI	p-value
Tumor location			
Left-sided	1.0	–	0.069
Right-sided	0.82	0.66–1.02	
Age			
< 83 years	1.0	–	0.12
≥ 83 years	1.18	0.95–1.46	
Sex			
Male	1.0	–	< 0.01
Female	0.68	0.55–0.84	
Body Mass Index			
< 18.5	1.0	–	0.32
18.5 to < 25.0	0.90	0.69–1.18	
≥ 25.0	1.11	0.78–1.58	
ASA-class			
1, 2	1.0	–	0.61
3, 4	0.93	0.72–1.21	
Comorbidity			
No comorbidity	1.0	–	0.40
Cardiovascular	1.23	0.94–1.61	0.14
Renal	1.81	0.90–3.63	0.094
Diabetes mellitus	0.96	0.69–1.33	0.81
Previous abdominal surgery			
Absent	1.0	–	0.12
Present	1.20	0.96–1.49	
TNM stage			
0–1	1.0	–	< 0.01
2	1.59	1.21–2.09	
3	1.45	1.09–1.94	
Approach			
Open	1.0	–	< 0.01
Laparoscopic	0.67	0.53–0.84	

*ASA* American Society of Anesthesiologists

### The comparison of long-term outcomes between RCCs and LCCs after curative surgery

**Fig. 2:**
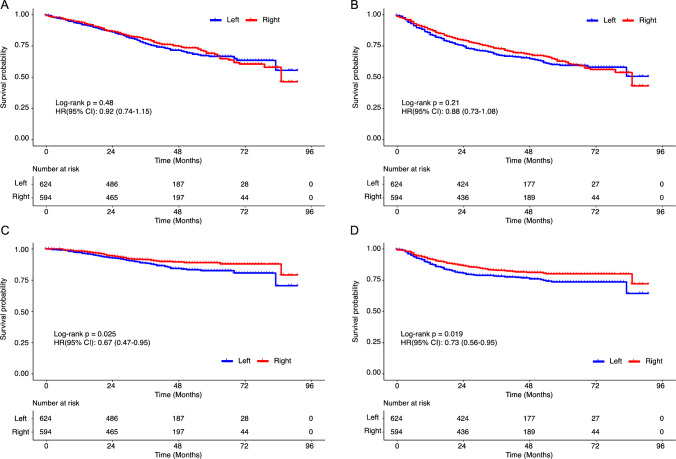
The comparison of survival outcomes between right- and left-sided colorectal cancers (pathological stage II–III). The overall survival (**A**), relapse-free survival (**B**), cancer-specific survival (**C**), and cancer-specific relapse-free survival (**D**). The data were summarized as hazard ratios (HRs) with 95% confidence intervals (CIs) and p-values based on a log-rank test

In survival analyses, there were no marked differences in the OS or RFS between the RCC and LCC groups (Fig. 2A and B). However, the RCC group showed a better CS-S and CS-RFS rates than the LCC group (Fig. 2C and D). Next, we investigated the effect of sidedness on the pathological stages II and III. Although there was a consistent tendency toward a better prognosis in RCCs than in LCCs, these differences were not statistically significant (Supplementary Fig. 1). However, in the multivariate analysis using a Cox proportional hazard model, tumor sidedness was determined to be an independent prognostic factor associated with the CS-S and CS-RFS, with both being more favorable in the RCC group than in the LCC group. In addition to tumor sidedness, a lower BMI and higher TNM stage (for the CS-S) and renal comorbidity and a higher TNM stage (for the CS-RFS) were determined to be independent prognostic factors in this cohort (Table 4).

**Table 4:** Results of a multivariate analysis for the cancer-specific overall and relapse-free survival in patients ≥ 80 years old with colorectal cancer who underwent curative surgery

	Cancer-specific survival			Cancer-specific relapse-free survival		
Factor	Hazard ratio	95% CI	p-value	Hazard ratio	95% CI	p-value
Tumor location			0.037			0.043
Left-sided	1.0	–		1.0	–	
Right-sided	0.68	0.47–0.98		0.76	0.58–0.99	
Age			0.37			0.21
< 83 years	1.0	–		1.0	–	
≥ 83 years	1.18	0.82–1.69		1.19	0.91–1.55	
Sex			0.52			0.14
Male	1.0	–		1.0	–	
Female	1.13	0.78–1.63		0.81	0.62–1.07	
Body Mass Index						
< 18.5	1.0	–		1.0	–	
18.5 to < 25.0	0.56	0.37–0.84	< 0.01	0.75	0.54–1.04	0.081
≥ 25.0	0.56	0.31–1.01	0.052	0.79	0.51–1.23	0.30
ASA-class			0.030			0.065
1, 2	1.0	–		1.0	–	
3, 4	1.58	1.05–2.40		1.34	0.98–1.83	
Comorbidity						
No comorbidity	1.0	–		1.0	–	
Cardiovascular	0.73	0.44–1.19	0.20	1.00	0.71–1.40	0.98
Renal	1.59	0.57–4.40	0.38	2.14	1.12–4.09	0.021
Diabetes mellitus	1.21	0.70–2.11	0.49	1.20	0.91–1.58	0.64
Previous abdominal surgery			0.67			0.20
Absent	1.0	–		1.0	–	
Present	0.92	0.63–1.35		1.20	0.91–1.58	
TNM Stage			< 0.01			< 0.01
II	1.0	–		1.0	–	
III	2.85	1.96–4.13		2.39	1.82–3.12	
Histology			0.91			0.24
Others	1.0	–		1.0	–	
Muc + Sig	0.95	0.39–2.34		0.61	0.27–1.39	
Approach			0.60			0.60
Open	1.0	–		1.0	–	
Laparoscopic	0.90	0.61–1.33		0.92	0.69–1.24	

*ASA* American Society of Anesthesiologists, *Muc + Sig* Mucinous adenocarcinoma and Signet ring cell carcinoma, *CI* confidence interval

### Frequency of MSI-high cases among the elderly by sidedness of CRC in the TCGA-COADREAD dataset

We next examined the impact of the MSI status on differences in the CS-S and CS-RFS in the elderly population. However, due to the study design and ethical review board, MSI status data and formalin-fixed paraffin-embedded blocks were unavailable. Therefore, instead of analyzing the MSI status in our cohort, we performed a public dataset analysis of the TCGA Cancer Atlas COADREAD dataset. Using this dataset, we analyzed the association between age, sex, and MSI status estimated using the MSIsensor and MSI MANTIS scores. MSI-high cases were more frequently observed among patients ≥ 80 years old with RCC than in those < 80 years old (Table 5).

**Table 5:** Frequency of microsatellite unstable CRCs by age, sex, and sidedness in TCGA pancancer atlas COADREAD dataset

	All	Male	Female
Age, ≥ 80 years			
Left	2/32 (6.3%)	1/15 (6.7%)	1/17 (5.9%)
Right	31/63 (49.2%)	10/29 (34.5%)	21/34 (61.8%)
Age, < 80 years			
Left	20/295 (6.8%)	9/156 (5.8%)	11/139 (7.9%)
Right	26/88 (29.5%)	28/112 (25.0%)	54/200 (27.0%)
Age, < 70 years			
Left	16/210 (7.6%)	7/116 (6.0%)	9/94 (9.6%)
Right	32/125 (25.6%)	18/72 (25.0%)	14/53 (26.4%)

## Discussion

Sidedness of CRC is reportedly associated with genomic instability [12, 14, 15, 23, 26–29]. MSI-high CRCs is predominantly observed in the right-sided colon and more frequently in elderly females than in males [14, 15, 31, 32]. Consistent with these reported features of MSI-high CRCs, there were more females in the RCC group than in the LCC group in the present study (Table 1). Furthermore, the ratio of RCCs to LCCs was 1:1 in our cohort, although it was reported to be 3:7 in the general population. This is also consistent with previous studies reporting that RCCs were more frequent among the elderly than among younger patients [39]. In addition, mucinous adenocarcinomas and signet-ring cell carcinomas, both of which are characteristic histological features of MSI-high CRCs [31, 32], were found more frequently in the RCC group than in the LCC group. Taken together, these findings suggest that the elderly population has more MSI-high CRCs in the right-sided colon than the general population. In addition, our TCGA dataset analysis showed that MSI-high cases were remarkably frequent in RCC cases among elderly patients ≥ 80 years old (Table 5), although we did not examine MSI in our cohort.

This high frequency of MSI-high CRC in the elderly may have affected the survival outcomes after curative surgery in our cohort because of the low potential for distant metastasis and recurrence. Although there were no significant differences in the OS or RFS between the RCC and LCC groups, the RCC group tended to have better survival than the LCC group according to the CS-S and CS-RFS (Fig. 2). In subsequent multivariate analyses, tumor sidedness and TNM stage were common independent risk factors for the CS-S and CS-RFS (Table 4). However, despite the better prognosis in the RCC group than in the LCC group in our cohort, others have reported that RCCs have a worse prognosis than LCCs after surgery [17, 23]. Furthermore, a recent meta-analysis including 15 independent studies concluded that RCCs had a worse OS than LCCs [24]. This discrepancy may be explained by the extremely high frequency of MSI-high CRC in patients who were ≥ 80 years old and had RCC. In our TCGA dataset analysis (Table 5), MSI-high CRC was more frequent in RCCs than in LCCs in all studied populations. Furthermore, MSI-high cases were much more frequent in patients ≥ 80 years old than in those < 80 years old. Although there are some issues regarding race and geographical differences in the TCGA dataset because of the worldwide nature of the TCGA Cancer Atlas project, the results from the TCGA dataset potentially support our conclusion that RCCs have a better prognosis than LCCs among elderly patients in Japan.

The definition of LCC remains controversial, although the present study also included the rectum. We included rectal cancers in the left-sided group based on a previous report that tumor sidedness simplifies the continuum in the mutational profile and consensus molecular subtype (CMS), which influences the OS. The relative prevalence of the CMS and MSI-high status is reportedly comparable among sigmoid, rectosigmoid, and rectal cancers [40]. Our results also indicated that the CS-S in rectal cancers from rectum/above and rectum/below the peritoneal reflection cancers, despite the fact that there were no cases of preoperative radiotherapy for middle and lower rectal cancers, were not inferior to those of cancers from other anatomic sites, as shown in Supplementary Table 2, indicating that the prognosis of LCC is not worse than that of RCC due to the inclusion of the rectum.

As the present study targeted an elderly population, we focused on the association between the pre-operative conditions and short-term outcomes. Regarding differences in tumor sidedness, surgeries for LCCs appeared to be more invasive than those for RCCs based on surgical duration, blood loss, incidence of complications, and length of hospital stay (Table 2). However, tumor location had no notable impact on the morbidity rate in the multivariate analysis. Furthermore, laparoscopic surgery reduced the risk of post-operative complications in both the RCC and LCC groups (Table 3 and Supplementary Fig. 2). Although care should be taken when dealing with male patients and those with a high TNM stage, a laparoscopic procedure can be safely chosen regardless of the tumor location (Table 3).

Several limitations associated with the present study warrant mention. First, because of the retrospective nature of the study, some biases could not be avoided, despite our efforts to reduce the impact of such biases using multivariate analyses. However, because all elderly patients who underwent surgery were enrolled during the investigation period (for five years, 2003–2007), and because there did not seem to be any extreme trends in the proportion of RCCs and LCCs among institutions (Supplementary Fig. 3), the results are thought to represent the current situation in Japan to some degree. Second, almost no patients received adjuvant chemotherapy for stage III disease after curative surgery. MSI-high CRCs have been reported to be resistant to 5-fluorouracil (5-FU), which may affect survival outcomes [35]. The combination of oxaliplatin and 5-FU was reported to improve the RFS in MSI-high CRCs among the general population [35], although no survival benefits were achieved by adding oxaliplatin to 5-FU-based adjuvant chemotherapy in the elderly population [41–43]. In addition, the latest clinical trial revealed a notable effect of a novel immune checkpoint inhibitor on CRCs with deficient mismatch repair in a neoadjuvant setting in MSI-high patients [44]. The optimum adjuvant chemotherapy regimen for elderly patients with CRC should be elucidated in future studies. The MSI status has increased in importance as a promising biomarker, so now might be an ideal time to consider the routine investigation of MSI in CRCs and the expansion of insurance coverage after curative surgery.

In conclusion, RCCs showed a better CS-S in elderly patients ≥ 80 years old than LCCs. Although the MSI status was not investigated, the results suggest that MSI-high CRC, which is predominantly seen in the elderly and in RCCs, may affect survival outcomes. Further analyses, including assessments of molecular aberrations, are needed to identify the prognostic factors of CRCs in both the elderly and general populations.

## Supplementary Information

Below is the link to the electronic supplementary material.

The comparison of survival outcomes between right- and left-sided colorectal cancers by pathological stage II and III. The cancer-specific survival for stage II (A), cancer-specific relapse-free survival for stage II (B), cancerspecific survival for stage III (C), and cancer-specific relapse-free survival for stage III (D). The data were summarized as hazard ratios (HRs) with 95% confidence intervals (CIs) and p-values based on a log-rank test. Supplementary file1 (DOCX 181 KB)

The comparison of the morbidities as postoperative complications in patients ≥80 years old between the open surgery and laparoscopic surgery groups for each type of CRC. Supplementary file2 (DOCX 47 KB)

Supplementary file3 (DOCX 70 KB)

Supplementary file4 (DOCX 16 KB)

Supplementary file5 (DOCX 16 KB)
