# Supplementary figures and images for: Right-sided versus left-sided colorectal cancer in elderly patients: a sub-analysis of a large multicenter case–control study in Japan
Source: Surg Today. 2024 Jun 5;54(10):1173–83. doi: 10.1007/s00595-024-02827-9 (PMC11413077; doi:10.1007/s00595-024-02827-9)

Supplementary Figure 3. Proportion of RCCs and LCCs among institutions


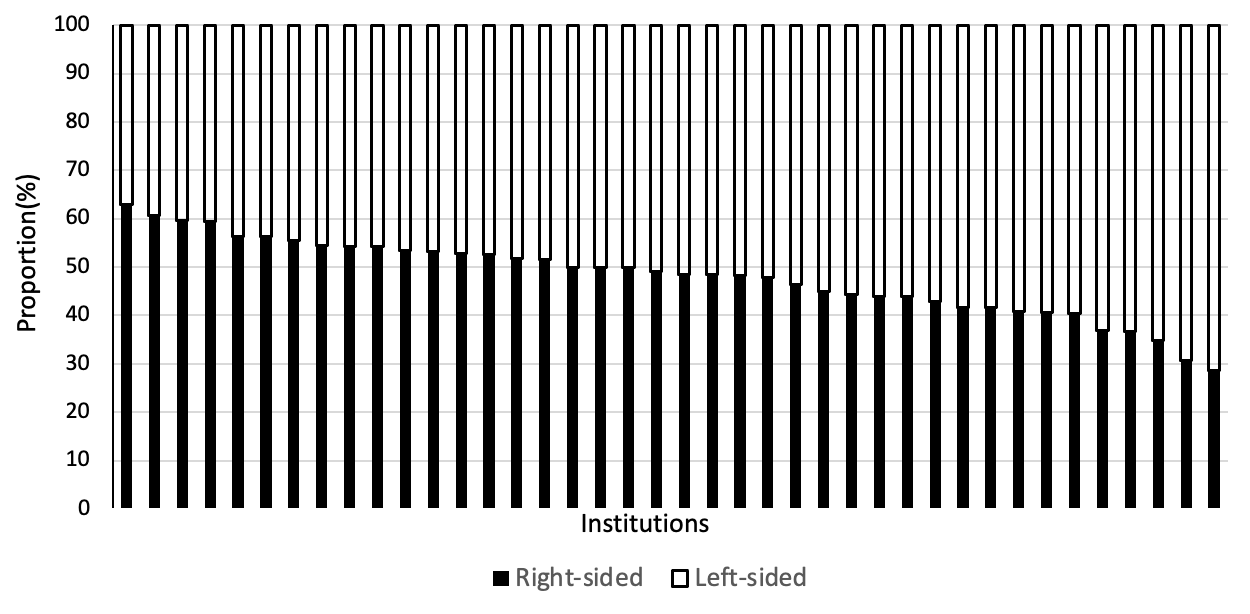

Supplement: Supplementary file 3 — Supplementary file3 (DOCX 70 KB) [file 595_2024_2827_MOESM3_ESM.docx]
