# Supplementary material for: Right-sided versus left-sided colorectal cancer in elderly patients: a sub-analysis of a large multicenter case–control study in Japan
Source: Surg Today. 2024 Jun 5;54(10):1173–83. doi: 10.1007/s00595-024-02827-9 (PMC11413077; doi:10.1007/s00595-024-02827-9)
Supplement: Supplementary file 4 — Supplementary file4 (DOCX 16 KB) [file 595_2024_2827_MOESM4_ESM.docx]

Supplementary Table 1. Tumor locations in patients ≥80 years old in this study

|  | n (%) | |
| --- | --- | --- |
| Cecum | 193 (11.5%) |  |
| Ascending | 405 (24.1%) | 812 (48.3%) |
| Transverse | 214 (12.7%) |  |
| Descending | 66 (3.9%) |  |
| Sigmoid | 375(22.3%) |  |
| upper rectum | 165 (9.8%) | 868 (51.7%) |
| middle rectum | 124 (7.4%) |  |
| lower rectum | 138 (8.2%) |  |
