# Supplementary material for: Right-sided versus left-sided colorectal cancer in elderly patients: a sub-analysis of a large multicenter case–control study in Japan
Source: Surg Today. 2024 Jun 5;54(10):1173–83. doi: 10.1007/s00595-024-02827-9 (PMC11413077; doi:10.1007/s00595-024-02827-9)
Supplement: Supplementary file 5 — Supplementary file5 (DOCX 16 KB) [file 595_2024_2827_MOESM5_ESM.docx]

Supplementary Table 2. Cox proportional hazard model for the cancer-specific survival based on tumor location relative to Ra+Rb

|  | | | | |
| --- | --- | --- | --- | --- |
|  | n | Hazard | 95% CI | p |
| Ra+Rb | 182 | 1.00 | reference | - |
| RS | 129 | 0.763 | 0.415-1.405 | 0.386 |
| Sigmoid | 216 | 0.687 | 0.409-1.154 | 0.156 |
| Descending | 52 | 0.627 | 0.243-1.620 | 0.335 |
| Transverse | 156 | 0.500 | 0.260-0.962 | 0.038 |
| Ascending | 308 | 0.622 | 0.375-1.032 | 0.066 |
| Cecum | 130 | 0.360 | 0.165-0.789 | 0.011 |

*CI: confidence interval, Ra: rectum/above the peritoneal reflection, Rb: rectum/below the peritoneal reflection, RS: recto-sigmoid*
